# Supplementary material for: Discovery and application of insertion-deletion (INDEL) polymorphisms for QTL mapping of early life-history traits in Atlantic salmon
Source: BMC Genomics. 2010 Mar 8;11:156. doi: 10.1186/1471-2164-11-156 (PMC2838853; doi:10.1186/1471-2164-11-156)
Supplement: Additional file 2 — Information on developed 76 locus single-run INDEL panel in Atlantic salmon. Information on fluorescence labeling, primer concentrations, PCR pooling and links to alignments, INDEL motifs and GENESCAN (Burge and Karlin 1997) predictions of genes/exons are available in html format. [file 1471-2164-11-156-S2.ZIP › Additionalfile2/table15900.html]

Contig Alignment

```
Base	A B C D E F G H   
1	G G G . G . . G    
2	T T T . T . . T    
3	A A A . A . . A    
4	G G G . G . . G    
5	C C C . C . . C    
6	T T T . T . . T    
7	T T T . T . . T    
8	T T T . T . . T    
9	T C C . C . . C    
10	G G G . G . . G    
11	T T T . T . . T    
12	T T T . T . . T    
13	T T T . T . . T    
14	A A A . A . . A    
15	T T T . T . . T    
16	T T T . T . . T    
17	T T T . T . . T    
18	T T T . T . . T    
19	G G G . G . . G    
20	A A A . A . . A    
21	A A A . A . . A    
22	C C C . C . . C    
23	C C C . C . . C    
24	A A A . A . . A    
25	T T T . T T T T    
26	A A A . A A A A    
27	A A A . A A A A    
28	G G G . G G G G    
29	T T T . T T T T    
30	T T T . T T T T    
31	T T T . T T T T    
32	C C C . C C C C    
33	A A A . A A A A    
34	A A A . A A A A    
35	A A A . A A A A    
36	A A A . A A A A    
37	T T T . T T T T    
38	A A A . A A A A    
39	A A A . A A A A    
40	T T T . T T T T    
41	C C C . C C C C    
42	T T T . T T T T    
43	C C C . C C C C    
44	T T T . T T T T    
45	T T T . T T T T    
46	A A A . A A A A    
47	T T T . T T T T    
48	T T T . T T T T    
49	C C C . C C C C    
50	A A A . A A A A    
51	A A A . A A A A    
52	C C C . C C C C    
53	A A A . A A A A    
54	C C C . C C C C    
55	C C C . C C C C    
56	A A A . A A A A    
57	A A A . A A A A    
58	T T T . T T T T    
59	G G G . G G G G    
60	G G G . G G G G    
61	T T T . T T T T    
62	A A A . A A A A    
63	T T T . T T T T    
64	T T T . T T T T    
65	G G G . G G G G    
66	T T T . T T T T    
67	A A A . A A A A    
68	A A A . A A A A    
69	A A A . A A A A    
70	A A A . A A A A    
71	C C C . C C C C    
72	A A A . A A A A    
73	A A A . A A A A    
74	A A A . A A A A    
75	A A A . A A A A    
76	- C C . C - - C  3	 indel  
77	- A A . A - - A  3	 indel  
78	- A A . A - - A  3	 indel  
79	- A A . A - - A  3	 indel  
80	- A A . A - - A  3	 indel  
81	A A A . A A A A    
82	T T T . T T T T    
83	C C C . C C C C    
84	C C C . C C C C    
85	T T T . T T T T    
86	G G G . G G G G    
87	T T T . T T T T    
88	G G G . G G G G    
89	C C C . C C C C    
90	A A A . A A A A    
91	A A A . A A A A    
92	A A A . A A A A    
93	C C C . C C C C    
94	T T T . T T T T    
95	G G G . G G G G    
96	T T T . T T T T    
97	G G G . G G G G    
98	A A A . A A A A    
99	A A A . A A A A    
100	T T T . T T T T    
101	T T T . T T T T    
102	G G G . G G G G    
103	A A A . A A A A    
104	T T T . T T T T    
105	T T T . T T T T    
106	T T T . T T T T    
107	A A A . A A A A    
108	A A A . A A A A    
109	G G G . G G G G    
110	T T T . T T T T    
111	A A A . A A A A    
112	C C C . C C C C    
113	A A A . A A A A    
114	C C C . C C C C    
115	C C C . C C C C    
116	C C C . C C C C    
117	T T T . T T T T    
118	T T T . T T T T    
119	C C C . C C C C    
120	A A A . A A A A    
121	A A A . A A A A    
122	T T T . T T T T    
123	A A A . A A A A    
124	T T T . T T T T    
125	A A A . A A A A    
126	A A A . A A A A    
127	A A A . A A A A    
128	T T T . T T T T    
129	A A A . A A A A    
130	T T T . T T T T    
131	G G G . G G G G    
132	G G G . G G G G    
133	C C C . C C C C    
134	C C C . C C C C    
135	T T T . T T T T    
136	A A A . A A A A    
137	T T T . T T T T    
138	A A A . A A A A    
139	T T T . T T T T    
140	G G G . G G G G    
141	C C C . C C C C    
142	T T T . T T T T    
143	T T T . T T T T    
144	T T T . T T T T    
145	C C C . C C C C    
146	A A A . A A A A    
147	T T T . T T T T    
148	A A A . A A A A    
149	A A A . A A A A    
150	A A A . A A A A    
151	C C C . C C C C    
152	T T T . T T T T    
153	A A A . A A A A    
154	A A A . A A A A    
155	C C C . C C C C    
156	C C C . C C C C    
157	C C C . C C C C    
158	C C C . C C C C    
159	T T T . T T T T    
160	T T T . T T T T    
161	T T T . T T T T    
162	A A A . A A A A    
163	C C C . C C C C    
164	A A A . A A A A    
165	T G G . G G G G    
166	T - - . - - - -    
167	C - - . - - - -    
168	C C C . C C C C    
169	A A A . A A A A    
170	G G G . G G G G    
171	T T T . T T T T    
172	G G G . G G G G    
173	A A A . A A A A    
174	T T T . T T T T    
175	A A A . A A A A    
176	T T T . T T T T    
177	C C C . C C C C    
178	A A A A A A A A    
179	C C C C C C C C    
180	A A A A A A A A    
181	G G G G G G G G    
182	C C C C C C C C    
183	T T T T T T T T    
184	T T T T T T T T    
185	C C C C C C C C    
186	T T T T T T T T    
187	G G G G G G G G    
188	A A A A A A A A    
189	A A A A A A A A    
190	A A A A A A A A    
191	A A A A A A A A    
192	C C C C C C C C    
193	G G G G G G G G    
194	A A A A A A A A    
195	A A A A A A A A    
196	G G G G G G G G    
197	C C C C C C C C    
198	C C C C C C C C    
199	C C C C C C C C    
200	A A A A A A A A    
201	G G G G G G G G    
202	T T T T T T T T    
203	G G G G G G G G    
204	T T T T T T T T    
205	G G G G G G G G    
206	T T T T T T T T    
207	T T T T T T T T    
208	T T T T T T T T    
209	G G G G G G G G    
210	T T T T T T T T    
211	C C C C C C C C    
212	A A A A A A A A    
213	A A A A A A A A    
214	G G G G G G G G    
215	A A A A A A A A    
216	T T T T T T T T    
217	T T T T T T T T    
218	C C C C C C C C    
219	C C C C C C C C    
220	A A A A A A A A    
221	A A A A A A A A    
222	A A A A A A A A    
223	T T T T T T T T    
224	T T T T T T T T    
225	A A A A A A A A    
226	A A A A A A A A    
227	A A A A A A A A    
228	T T T T T T T T    
229	T T T T T T T T    
230	A A A A A A A A    
231	T T T T T T T T    
232	C C C C C C C C    
233	T T T T T T T T    
234	G G G G G G G G    
235	C C C C C C C C    
236	A A A A A A A A    
237	C C C C C C C C    
238	C C C C C C C C    
239	C C C C C C C C    
240	C C C C C C C C    
241	A A A A A A A A    
242	T T T T T T T T    
243	C C C C C C C C    
244	A A A A A A A A    
245	C C C C C C C C    
246	T T T T T T T T    
247	T T T T T T T T    
248	G G G G G G G G    
249	A A A A A A A A    
250	C C C C C C C C    
251	A A A A A A A A    
252	C C C C C C C C    
253	C C C C C C C C    
254	A A A A A A A A    
255	- - - A - - - -    
256	T T T T T T T T    
257	T T T T T T T T    
258	G G G G G G G G    
259	A A A A A A A A    
260	G G G G G G G G    
261	C C C C C C C C    
262	T T T T T T T T    
263	G G G G G G G G    
264	A A A A A A A A    
265	A A A A A A A A    
266	A A A A A A A A    
267	A A A A A A A A    
268	G G G G G G G G    
269	A A A A A A A A    
270	C C C C C C C C    
271	A A A A A A A A    
272	A G G G G G G G    
273	A A A A A A A A    
274	C C C C C C C C    
275	A A A A A A A A    
276	T T T T T T T T    
277	T T T T T T T T    
278	T T T T T T T T    
279	T T T T T T T T    
280	T T T T T T T T    
281	A A A A A A A A    
282	C C C C C C C C    
283	C C C C C C C C    
284	A A A A A A A A    
285	T T T T T T T T    
286	A A A A A A A A    
287	C C C C C C C C    
288	T T T T T T T T    
289	G G G G G G G G    
290	T T T T T T T T    
291	A A A A A A A A    
292	G G G G G G G G    
293	C C C C C C C C    
294	T T T T T T T T    
295	C C C C C C C C    
296	T T T T T T T T    
297	G G G G G G G G    
298	A A A A A A A A    
299	G G G G G G G G    
300	C C C C C C C C    
301	C C C C C C C C    
302	C C C C C C C C    
303	T T T T T T T T    
304	G G G G G G G G    
305	T T T T T T T T    
306	G G G G G G G G    
307	T T T T T T T T    
308	G G G G G G G G    
309	G G G G G G G G    
310	A A A A A A A A    
311	T T T T T T T T    
312	T T T T T T T T    
313	C C C C C C C C    
314	A A A A A A A A    
315	G G G G G G G G    
316	C C C C C C C C    
317	T T T T T T T T    
318	G G G G G G G G    
319	A A A A A A A A    
320	T T T T T T T T    
321	G G G G G G G G    
322	T T T T T T T T    
323	G G G G G G G G    
324	G G G G G G G G    
325	G G G G G G G G    
326	C C C C C C C C    
327	T T T T T T T T    
328	G G G G G G G G    
329	G G G G G G G G    
330	A A A A A A A A    
331	G G G G G G G G    
332	T T T T T T T T    
333	G G G G G G G G    
334	T T T T T T T T    
335	G G G G G G G G    
336	T T T T T T T T    
337	C C C C C C C C    
338	T T T T T T T T    
339	C C C C C C C C    
340	C C C C C C C C    
341	A A A A A A A A    
342	G G G G G G G G    
343	T T T T T T T T    
344	G G G G G G G G    
345	A A A A A A A A    
346	T T T T T T T T    
347	A A A A A A A A    
348	A A A A A A A A    
349	A G G G G G G G    
350	A A A A A A A A    
351	C C C C C C C C    
352	A A A A A A A A    
353	A A A A A A A A    
354	T T T T T T T T    
355	A A A A A A A A    
356	A A A A A A A A    
357	A A A A A A A A    
358	T T T T T T T T    
359	A A A A A A A A    
360	T T T T T T T T    
361	T T T T T T T T    
362	G G G G G G G G    
363	C C C C C C C C    
364	A A A A A A A A    
365	C C C C C C C C    
366	C C C C C C C C    
367	A A A A A A A A    
368	T T T T T T T T    
369	T T T T T T T T    
370	T G G G G G G G    
371	A A A A A A A A    
372	A A A A A A A A    
373	G G G G G G G G    
374	C C C C C C C C    
375	T T T T T T T T    
376	T T T T T T T T    
377	T T T T T T T T    
378	G G G G G G G G    
379	A A A A A A A A    
380	T T T T T T T T    
381	A A A A A A A A    
382	A A A A A A A A    
383	A A A A A A A A    
384	T T T T T T T T    
385	G G G G G G G G    
386	C C C C C C C C    
387	T T T T T T T T    
388	G G G G G G G G    
389	C C C C C C C C    
390	C C C C C C C C    
391	T T T T T T T T    
392	G G G G G G G G    
393	T T T T T T T T    
394	T T T T T T T T    
395	C C C C C C C C    
396	T T T T T T T T    
397	G G G G G G G G    
398	C C C C C C C C    
399	A A A A A A A A    
400	A A A A A A A A    
401	T T T T T T T T    
402	A A A A A A A A    
403	A A A A A A A A    
404	A A A A A A A A    
405	G G G G G G G G    
406	G G G G G G G G    
407	C C C C C C C C    
408	C A A A A A A A    
409	G G G G G G G G    
410	A A A A A A A A    
411	A A A A A A A A    
412	C C C C C C C C    
413	A A A A A A A A    
414	A A A A A A A A    
415	A A A A A A A A    
416	G G G G G G G G    
417	G G G G G G G G    
418	A A A A A A A A    
419	T T T T T T T T    
420	A A A A A A A A    
421	C C C C C C C C    
422	A A A A A A A A    
423	A A A A A A A A    
424	A A A A A A A A    
425	G G G G G G G G    
426	A A A A A A A A    
427	A A A A A A A A    
428	A A A A A A A A    
429	C C C C C C C C    
430	T T T T T T T T    
431	A A A A A A A A    
432	A A A A A A A A    
433	A A A A A A A A    
434	G G G G G G G G    
435	T T T T T T T T    
436	A A A A A A A A    
437	A A A A A A A A    
438	A A A A A A A A    
439	A A A A A A A A    
440	A A A A A A A A    
441	. A A A A A A A    
442	. A A A A A A A    
443	. G G G G G G G    
444	. A A A A A A A    
445	. A A A A A A A    
446	. G G G G G G G    
447	. C C C C C C C    
448	. G G G G G G G    
449	. G G G G G G G    
450	. T T T T T T T    
451	. G G G G G G G    
452	. A A A A A A A    
453	. C C C C C C C    
454	. A A A A A A A    
455	. A A A A A A A    
456	. T T T T T T T    
457	. C C C C C C C    
458	. A A A A A A A    
459	. A A A A A A A    
460	. A A A A A A A    
461	. G G G G G G G    
462	. T T T T T T T    
463	. A A A A A A A    
464	. A A A A A A A    
465	. T T T T T T T    
466	. T T T T T T T    
467	. A A A A A A A    
468	. T T T T T T T    
469	. G G G G G G G    
470	. A A A A A A A    
471	. C C C C C C C    
472	. A A A A A A A    
473	. G G G G G G G    
474	. G G G G G G G    
475	. T T T T T T T    
476	. G G G G G G G    
477	. T T T T T T T    
478	. C C C C C C C    
479	. T T T T T T T    
480	. G G G G G G G    
481	. T T T T T T T    
482	. C C C C C C C    
483	. T T T T T T T    
484	. A A A A A A A    
485	. C C C C C C C    
486	. A A A A A A A    
487	. C C C C C C C    
488	. A A A A A A A    
489	. T T T T T T T    
490	. A A A A A A A    
491	. G G G G G G G    
492	. G G G G G G G    
493	. G G G G G G G    
494	. T T T T T T T    
495	. A A A A A A A    
496	. A A A A A A A    
497	. G G G G G G G    
498	. C C C C C C C    
499	. A A A A A A A    
500	. G G G G G G G    
501	. A A A A A A A    
502	. G G G G G G G    
503	. G G G G G G G    
504	. C C C C C C C    
505	. A A A A A A A    
506	. T T T T T T T    
507	. A A A A A A A    
508	. C C C C C C C    
509	. A A A A A A A    
510	. C C C C C C C    
511	. C C C C C C C    
512	. A A A A A A A    
513	. T T T T T T T    
514	. T T T T T T T    
515	. C C C C C C C    
516	. C C C C C C C    
517	. T T T T T T T    
518	. C C C C C C C    
519	. C C C C C C C    
520	. A A A A A A A    
521	. C C C C C C C    
522	. A A A A A A A    
523	. C C C C C C C    
524	. A A A A A A A    
525	. A A A A A A A    
526	. T T T T T T T    
527	. A A A A A A A    
528	. A A A A A A A    
529	. A A A A A A A    
530	. T T T T T T T    
531	. A A A A A A A    
532	. C C C C C C C    
533	. A A A A A A A    
534	. C C C C C C C    
535	. A A A A A A A    
536	. T T T T T T T    
537	. T T T T T T T    
538	. C C C C C C C    
539	. C C A A A A A    
540	. . . C C C C C    
541	. . . T T T T T    
542	. . . A A A A A    
543	. . . A A A A A    
544	. . . T T T T T    
545	. . . C C C C C    
546	. . . A A A A A    
547	. . . T T T T T    
548	. . . G G G G G    
549	. . . C C C C C    
550	. . . A A A A A    
551	. . . C C C C C    
552	. . . C C C C C    
553	. . . C C C C C    
554	. . . A A A A A    
555	. . . A A A A A    
556	. . . A A A A A    
557	. . . C C C C C    
558	. . . G G G G G    
559	. . . G G G G G    
560	. . . C C C C C    
561	. . . A A A A A    
562	. . . A A A A A    
563	. . . A A A A A    
564	. . . C C C C C    
565	. . . A A A A A    
566	. . . G G G G G    
567	. . . A A A A A    
568	. . . G G G G G    
569	. . . C C C C C    
570	. . . C C C C C    
571	. . . G G G G G    
572	. . . C C C C C    
573	. . . T T T T T    
574	. . . A A A A A    
575	. . . G G G G G    
576	. . . T T T T T    
577	. . . A A A A A    
578	. . . C C C C C    
579	. . . A A A A A    
580	. . . C C C C C    
581	. . . A A A A A    
582	. . . A A A A A    
583	. . . A A A A A    
584	. . . C C C C C    
585	. . . A A A A A    
586	. . . A A A A A    
587	. . . A A A A A    
588	. . . T T T T T    
589	. . . T T T T T    
590	. . . A A A A A    
591	. . . A A A A A    
592	. . . A A A A A    
593	. . . T T T T T    
594	. . . T T T T T    
595	. . . G G G G G    
596	. . . C C C C C    
597	. . . A A A A A    
598	. . . T T T T T    
599	. . . C C C C C    
600	. . . A A A A A    
601	. . . G G G G G    
602	. . . G G G G G    
603	. . . A A A A A    
604	. . . T T T T T    
605	. . . A A A A A    
606	. . . A A A A A    
607	. . . T T T T T    
608	. . . G G G G G    
609	. . . T T T T T    
610	. . . G G G G G    
611	. . . C C A A A    
612	. . . . . C C C    
613	. . . . . A A A    
614	. . . . . G G G    
615	. . . . . G G G    
616	. . . . . A A A    
617	. . . . . A A A    
618	. . . . . C C C    
619	. . . . . G G G    
620	. . . . . G G G    
621	. . . . . A A A    
622	. . . . . A A A    
623	. . . . . G G G    
624	. . . . . T T T    
625	. . . . . G G G    
626	. . . . . A A A    
627	. . . . . A A A    
628	. . . . . A A A    
629	. . . . . C C C    
630	. . . . . T T T    
631	. . . . . C C C    
632	. . . . . C C C    
633	. . . . . A A A    
634	. . . . . C C C    
635	. . . . . T T T    
636	. . . . . C C C    
637	. . . . . A A A    
638	. . . . . A A A    
639	. . . . . G G G    
640	. . . . . T T T    
641	. . . . . A A A    
642	. . . . . C C C    
643	. . . . . A A A    
644	. . . . . G G G    
645	. . . . . A A A    
646	. . . . . G G G    
647	. . . . . A A A    
648	. . . . . A A A    
649	. . . . . T T T    
650	. . . . . C C C    
651	. . . . . T T T    
652	. . . . . A A A    
653	. . . . . T T T    
654	. . . . . A A A    
655	. . . . . C C C    
656	. . . . . A A A    
657	. . . . . T T T    
658	. . . . . A A A    
659	. . . . . T T T    
660	. . . . . G G G    
661	. . . . . A A A    
662	. . . . . G G G    
663	. . . . . C C C    
664	. . . . . T T T    
665	. . . . . A A A    
666	. . . . . A A A    
667	. . . . . T T T    
668	. . . . . A A A    
669	. . . . . C C C    
670	. . . . . A A A    
671	. . . . . G G G    
672	. . . . . T T T    
673	. . . . . A A A    
674	. . . . . T T T    
675	. . . . . G G G    
676	. . . . . T T T    
677	. . . . . A A A    
678	. . . . . C C C    
679	. . . . . C C C    
680	. . . . . C C C    
681	. . . . . T T T    
682	. . . . . A A A    
683	. . . . . A A A    
684	. . . . . A A A    
685	. . . . . C C C    
686	. . . . . A A A    
687	. . . . . G G G    
688	. . . . . T T T    
689	. . . . . A A A    
690	. . . . . A A A    
691	. . . . . C C C    
692	. . . . . G G G    
693	. . . . . G G G    
694	. . . . . T T T    
695	. . . . . T T T    
696	. . . . . T T T    
697	. . . . . C C C    
698	. . . . . A A A    
699	. . . . . C C C    
700	. . . . . T T T    
701	. . . . . A A A    
702	. . . . . T T T    
703	. . . . . C C C    
704	. . . . . A A A    
705	. . . . . T T T    
706	. . . . . C C C    
707	. . . . . C C C    
708	. . . . . T T T    
709	. . . . . C C C    
710	. . . . . G G G    
711	. . . . . A A A    
712	. . . . . A A A    
713	. . . . . C C C    
714	. . . . . C C C    
715	. . . . . T T T    
716	. . . . . G G G    
717	. . . . . C C C    
718	. . . . . T T T    
719	. . . . . C C C    
720	. . . . . G G G    
721	. . . . . T T T    
722	. . . . . C C C    
723	. . . . . T T T    
724	. . . . . A A A    
725	. . . . . T . T    
726	. . . . . T . T    
727	. . . . . C . C    
728	. . . . . A . A    
729	. . . . . C . C    
730	. . . . . A . A    
731	. . . . . T . T    
732	. . . . . T . T    
733	. . . . . G . G    
734	. . . . . A . A    
735	. . . . . T . T    
736	. . . . . T . T    
737	. . . . . C . C    
738	. . . . . A . A    
739	. . . . . A . A    
740	. . . . . T . T    
741	. . . . . T . T    
742	. . . . . C . C    
743	. . . . . T . T    
744	. . . . . G . G    
745	. . . . . T . T    
746	. . . . . G . G    
747	. . . . . C . C    
748	. . . . . A . A    
749	. . . . . C . C    
750	. . . . . T . T    
751	. . . . . A . A    
752	. . . . . A . A    
753	. . . . . T . T    
754	. . . . . T . T    
755	. . . . . G . G    
756	. . . . . G . G    
757	. . . . . A . A    
758	. . . . . A . A    
759	. . . . . A . A    
760	. . . . . . . A    
761	. . . . . . . G    
762	. . . . . . . A    
763	. . . . . . . C    
764	. . . . . . . A    
765	. . . . . . . G    
766	. . . . . . . T    
767	. . . . . . . A    
768	. . . . . . . A    
769	. . . . . . . T    
770	. . . . . . . T    
771	. . . . . . . T    
772	. . . . . . . A    
773	. . . . . . . G    
774	. . . . . . . G    
775	. . . . . . . G    
776	. . . . . . . A    
777	. . . . . . . C    
778	. . . . . . . T    
779	. . . . . . . T    
780	. . . . . . . T    
781	. . . . . . . C    
782	. . . . . . . T    
783	. . . . . . . T    
784	. . . . . . . T    
785	. . . . . . . A    
786	. . . . . . . A    
787	. . . . . . . A    
788	. . . . . . . A    
789	. . . . . . . A    
790	. . . . . . . T    
791	. . . . . . . G    
792	. . . . . . . T    
793	. . . . . . . G    
794	. . . . . . . T    
795	. . . . . . . T    
796	. . . . . . . T    
797	. . . . . . . T    
798	. . . . . . . T    
799	. . . . . . . G    
800	. . . . . . . A    
801	. . . . . . . T    
802	. . . . . . . T    
803	. . . . . . . G    
804	. . . . . . . A    
805	. . . . . . . C    
806	. . . . . . . T    
807	. . . . . . . A    
808	. . . . . . . A
```
